# Evaluation of the Relationship Between Pulmonary Artery Hypertension and Esophageal Varices Bleeding in Transplantation Candidates

**DOI:** 10.7759/cureus.13355

**Published:** 2021-02-15

**Authors:** Nergis Ekmen, Sami Cifci

**Affiliations:** 1 Gastroenterology, Gazi University Faculty of Medicine, Ankara, TUR; 2 Gastroenterology, Başakşehir Çam and Sakura City Hospital, Istanbul, TUR

**Keywords:** bleeding, esophageal varices, liver transplantation, portal hypertension, pulmonary hypertension

## Abstract

Introduction: Esophageal varices bleeding (EVB) in liver cirrhosis is an important cause of mortality and morbidity. We aimed to study the relationship between systolic pulmonary artery pressure (sPAP) and EV grade and EVB.

Methods: A total of 229 patients, 183 male and 46 female, who were determined to have EV in the upper gastrointestinal tract endoscopy and who had a transthoracic echocardiogram (TTE) were included in this study.

Results: The frequency of pulmonary hypertension (PHT) and EVB was determined to be 16% and 45%, respectively, in our study, and 20% of those who had bleeding had PHT; 70.3% of the cases with PHT were determined to have grade III varices while this rate was lower at 52.9% in cirrhosis without PHT. A significant correlation was determined between Model for End-Stage Liver Disease (MELD) score, Child-Turcotte-Pugh score, platelet, albumin, and sPAP in those without a history of bleeding (p<0.05).

Conclusion: An increase in the rate of grade III varices has been noted along with the prevalence of PHT in patients with portal hypertension. It has been determined that the increase in PAP is associated with an increase in the MELD score, which is closely associated with mortality and morbidity. Therefore, this positive relationship between the MELD score and PHT may lead to an increase in the frequency of advanced-stage EV.

## Introduction

Liver cirrhosis (LC) is caused by long-term or repeated damages to liver tissues due to one or more reasons. These repeated damages cause an increase in intrahepatic vascular pressure along with the formation of generalized hepatocyte necrosis, fibrous tissue hyperplasia, and regenerative nodules. Portal hypertension (PH) occurring due to increased intrahepatic vascular resistance in cirrhosis is observed due to the combined effect of the damage occurring in hepatic sinusoids in addition to the disruption of the balance between vasodilator and vasoconstrictor substances [1].

Numerous spontaneous portosystemic shunts and esophageal varices (EV) may occur as a complication of long-term PH in patients with LC [2]. The prevalence of gastroesophageal varices in LC is approximately 50% and it is associated with the severity of the liver disease [3]. Varices bleeding occurs at a rate of 5% annually and it is associated with a high mortality rate (15%-25% in six weeks) [3].

Heart performance and liver function are closely related. The dysfunction of one organ often leads to functional impairment in the other organ [4]. When considered together with right ventricular function, pulmonary hypertension (PHT) is seen as an important cause of morbidity and mortality in LC and is associated with the pulmonary effects of LC. Porto-pulmonary hypertension (PPHT) is one of the two most common pulmonary complications in LC. PPHT is a life-threatening respiratory complication that develops following portal hypertension, which may lead to right ventricular failure as a result of increased resistance to pulmonary blood flow and is associated with a high mortality rate in advanced stage liver disease [5,6]. It is stated that PPHT, which progresses with the presence of portosystemic shunts, develops as a result of endothelial dysfunction with an imbalance between vasoconstrictive and vasodilator substances in the pulmonary circulation [7]. PPHT is defined as the mean pulmonary artery pressure (mPAP) higher than 25 mmHg in right heart catheterization and is common in cirrhotic patients. Moderate and severe PPHT (mPAP 35 and 45 mmHg, respectively) are less common and more serious [8,9].

An important factor causing PHT is chronic and/or acute pulmonary embolism (PE). Since there are studies indicating that the risk of venous thromboembolism (VTE) in patients with cirrhosis is twice as high in non-cirrhotic patients, this should not be ignored [10]. Low levels of protein C and antithrombin due to decreased synthesis capacity for thrombosis etiology are included among the reasons [11]. Furthermore, a study conducted by Northup et al. indicating that the risk of VTE is significantly higher in patients with cirrhosis demonstrated that a low level of serum albumin is an independent risk factor for PE and VTE [12].

There are many cardiac pathologies that can lead to liver disease; however, the common physiology for most of them is chronically high central venous pressures. The long-term increase in these pressures may clinically manifest as cardiac cirrhosis [13]. Hyper-dynamic circulation status and increased cardiac output in LC cause further increased wall stress in the pulmonary circulation. Ultimately, pulmonary vascular resistance is further increased due to vasoconstriction, progressive pulmonary remodeling, and thrombosis [14]. Chronic right ventricular tension and increased right atrial pressures may lead to progressive liver injury, EV, and splenomegaly [15].

We tried to evaluate the effects of sPAP, which is an indicator of right heart pressure, on esophageal varices bleeding (EVB).

## Materials and methods

Patients

This study was carried out between 2016 and 2019 by scanning the files of the patients with cirrhosis listed for liver transplantation at the liver transplantation center gastroenterology outpatient clinic, online data of the hospital, and their endoscopy records. It was planned to include the patients who had EV detected in upper gastrointestinal tract endoscopy and who had transthoracic echocardiogram (TTE) in the study. Patients under 18 years of age and those without EV detected in endoscopy were excluded from the study. A total of 296 patient files were scanned retrospectively; 55 patients without echocardiography and 12 patients below the age of 18 were excluded, and 229 patients were included in the study. The ages, genders, cirrhosis etiologies, systolic PAP (sPAP) in TTE, varices grade in endoscopy, EV bleeding histories in the last year, and laboratory values of the patients were recorded. Model for End-Stage Liver Disease (MELD) and Child-Turcotte-Pugh (CP) classification scores were calculated by evaluating the clinical information and laboratory results over recorded patient files.

Pulmonary artery pressures and endoscopy

sPAP values estimated according to the measurements made based on the recommendations of the American Echocardiography Association [16] and from the tricuspid regurgitation flow rate were recorded. The sPAP corresponding to mPAP >25 mmHg was accepted to be >38 mmHg PHT [17]. Endoscopies were performed by a gastroenterologist using Fujinon EG-580RD (Fujifilm, Düsseldorf, Germany) gastroscopy device. Varices grading was defined as grade I-III [18].

Statistical analysis

The data were analyzed using Statistical Package for the Social Sciences, version 21 (SPSS Inc., Chicago, IL). The conformance of continuous variables to normal distribution was evaluated using visual (histogram and probability plots) and analytical (Kolmogorov-Smirnov/Shapiro-Wilk tests) methods. In the part of the descriptive statistics, categorical variables are provided in numbers and percentages, data conforming to normal distribution are provided with arithmetic mean and standard deviations, and data not conforming to normal distribution are provided with median (minimum-maximum) values. The continuous variables between the independent groups were analyzed by independent Student's t-test and Mann-Whitney U test, whichever is appropriate. The comparison analysis for categorical variables between independent groups was carried out using the Pearson chi-square (χ^2^) test. Spearman's correlation analysis was carried out for the relationship between PAP, laboratory data, MELD score, and CP score. A p-value of <0.05 was considered to be statistically significant.

## Results

A total of 229 patients, 183 (79.9%) male and 46 (20.1%) female, were included in the study. The mean age of the whole group was 53.58±8.93 years. There was no statistically significant difference in the mean age between male (n=281, 53.04±9.29) and female (n=78, 52.54±11.17) (p=0.690). The prevalence of EVB and PHT in our study group was 45% and 16%, respectively. EVB was observed in 20% of cases with PHT. Clinico-demographic, laboratory and echocardiographic findings according to the presence of PHT are summarized in Table 1. According to this analysis, the age distribution between the groups was similar (p=0.513). The mean ejection fraction in the groups with and without PHT was 61.81±4.53 and 62.48±5.20, respectively (p=0.467). The most common etiological factor was determined to be hepatitis B. Grade III EV were detected in 70.3% of patients with PHT and in 52.9% of patients without PHT. However, no statistically significant difference was determined between the groups in terms of EV grade (p=0.343). While the history of varices bleeding was 48.6% in cases with PHT and it was lower in those without PHT at 40.1%, this difference was not statistically significant. The analysis carried out in terms of MELD score revealed a statistically significant difference between the groups (p=0.007), and MELD score was higher in the PHT group compared to those without PHT, but there was no difference in terms of the CP score. The levels of albumin and platelet were lower in the group with PHT, but the difference was not statistically significant.

**Table 1:** Comparison of demographic, clinical, and laboratory findings according to pulmonary hypertension in the study population PHT, pulmonary hypertension; EF, ejection fraction; TR, tricuspid regurgitation; MR, mitral regurgitation; NASH, nonalcoholic steatohepatitis; PSC, primary sclerosing cholangitis; PBC, primary biliary cholangitis; MELD, Model for End-Stage Liver Disease; AST, aspartate aminotransferase; ALT, alanine aminotransferase; GGT, gamma glutamyltranspeptidase; NA, not available *Mean±standard deviation ^a^Independent sample t-test ^b^Chi-square analysis ^c^Mann-Whitney U test

Variables	Normal, n=192	PHT, n=37	p
Age, years*	53.41±9.17	54.46±7.61	0.513^a^
Gender, n (%)			
Male	153 (79.7)	30 (81.1)	0.846^b^
Female	39 (20.3)	7 (18.9)	
Echocardiographic finding			
EF %	62.48±5.20	61.81±453	0.467^a^
TR, n (%)			
Mild	16 (34.8)	30 (65.2)	<0.001^a^
Moderate	0 (0)	7 (100)	
MR, n (%)			
Mild	16 (66.7)	8 (33.3)	
Moderate	2 (40)	3 (60)	
Right ventricular and atrial dilation, n (%)	4 (36.4)	7 (63.6)	
Left ventricular hypertrophy, n (%)	13 (76.5)	4 (23.5)	
Etiology, n (%)			
Hepatitis B	75 (39.1)	20 (54.1)	NA
Hepatitis C	28 (14.6)	5 (13.5)	
Cryptogenic	30 (15.6)	2 (5.4)	
Autoimmune hepatitis	2 (1)	1 (2.7)	
NASH	19 (9.9)	3 (8.1)	
Alcohol	32 (16.7)	4 (10.8)	
PSC	2 (1)	1 (2.7)	
PBC	2(1)	1 (2.7)	
Wilson disease	2 (1)	0 (0.0)	
Esophageal varices, n (%)			
Grade I	18 (9.4)	2 (5.4)	0.334^b^
Grade II	72 (37.7)	9 (24.3)	
Grade III	101 (52.9)	26 (70.3)	
Bleeding history, n (%)			
Yes	77 (40.1)	18 (48.6)	0.657^b^
No	115 (59.9)	19 (51.4)	
MELD score	15 (6-33)	18 (8-28)	0.003^c^
Child-Turcotte-Pugh score	9 (5-14)	10 (5-15)	0.243^c^
AST, U/L	59 (17-463)	52 (17-463)	0.683^c^
ALT, U/L	32 (7-100)	36 (9-144)	0.782^c^
GGT, U/L	48 (7-578)	40 (3-386)	0.198^c^
Albumin, gr/dL	2.92 (1.9-4.8)	2.9 (1.9-4.2)	0.918^c^
Platelet count (x10^9^/L)	74.5 (16-225)	68 (24-190)	0.270^c^

**Figure 1:**
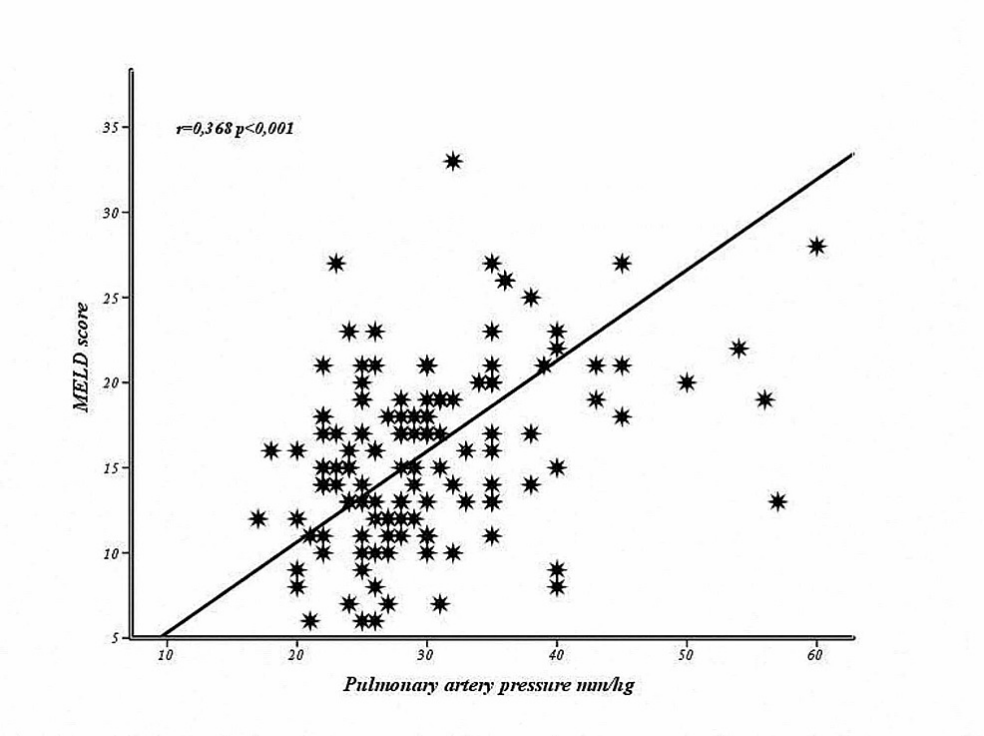
The relationship between pulmonary artery pressure and MELD score MELD, Model for End-Stage Liver Disease

**Figure 2:**
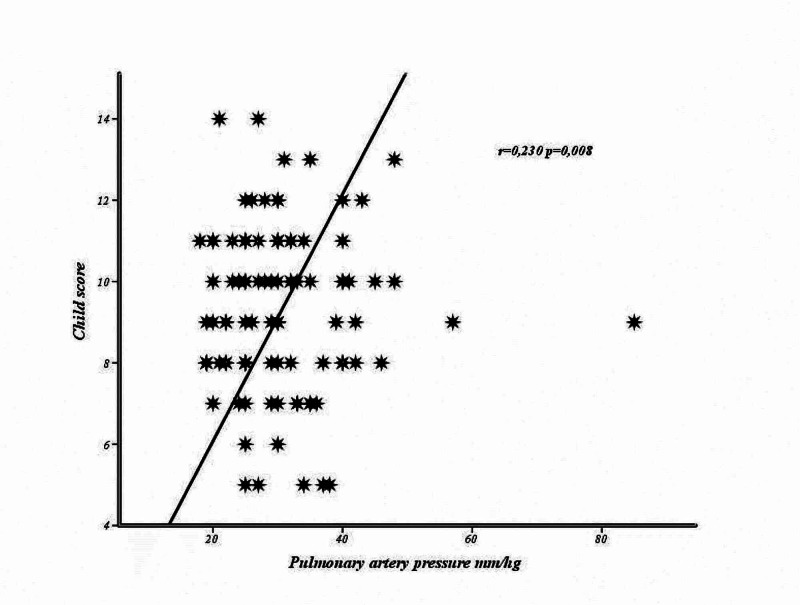
The relationship between pulmonary artery pressure and Child-Turcotte-Pugh score

The correlation analysis carried out between sPAP and laboratory parameters, CP and MELD scores according to bleeding status is summarized in Table 2. There was no correlation between sPAP and the parameters evaluated in cases who had bleeding (p>0.05). In cases without a history of bleeding, a significant correlation was determined between PAP and albumin, platelet, CP (Figure 1) and MELD scores (Figure 2) (p<0.05).

**Table 2:** Relationship of pulmonary artery pressure with laboratory parameters and MELD and Child scores according to bleeding status Spearman correlation analysis was used; Child score is the Child-Turcotte-Pugh score. MELD, Model for End-Stage Liver Disease; sPAP, systolic pulmonary artery pressure; AST, aspartate aminotransferase; ALT, alanine aminotransferase; GGT, gamma glutamyl transpeptidase

	Bleeding history			
Variables	Yes, n=95		No, n=134	
sPAP, mmHg	R	p	R	p
AST, U/L	-0.030	0.775	0.009	0.920
ALT, U/L	-0.003	0.978	0.033	0.703
Albumin, gr/dL	-0.027	0.798	-0.175	0.043
GGT, U/L	-0.024	0.815	-0.128	0.140
Platelet count	-0.123	0.234	-0.224	0.009
MELD score	0.073	0.482	0.368	<0.001
Child score	-0.010	0.920	0.230	0.008

## Discussion

The liver usually has a fragile balance with low-pressure blood flow and is easily affected by pressure changes. Any factor affecting drainage from the liver to the caval system will contribute to the disruption of this balance. As a result, the pressure increase in the caval vein, and the hepatic vein as a reflection of this may cause tissue damage. In an animal model where the caval vein was clamped, obstruction was shown to trigger liver fibrosis without a clear inflammatory process [19] . Similarly, right heart failure may cause a condition that can lead to liver congestion over time and eventually cause liver fibrosis. This condition may be the result of increased static pressure of perisinusoidal cells and tensile strength [20].

Portal hypertension and related complications may progress with the contribution of a hyper-dynamic syndrome characterized by an increase in right cardiac pressures in LC [19]. The analysis of the literature according to our information revealed no studies examining the relationship between the measurement of right heart pressure and EVB. Taking into account the entire pathophysiological process, we tried to examine the relationship between sPAP measured using the TTE method and EVB in our study.

We observed in our study that the prevalence of PHT was 16% and that the prevalence was high in patients with cirrhosis and PH findings in line with the literature [21]. In our study group, the prevalence of EVB was 45%, and 20% of these EVB patients had PHT. However, the effect of PHT on EVB was not statistically significant (p=0.657). Grade III varices were detected in 70.3% of patients with PHT. However, this rate was lower with 52.9% in cirrhosis without PHT. However, no statistically significant difference was determined between the groups in terms of EV grade (p=0.343). While the history of varices bleeding was 48.6% in cases with PHT and it was lower in those without PHT at 40.1%, this difference was not statistically significant. Considering the relationship between varices size and bleeding, it is evident that there is a need for larger-scale studies where PHT is classified as mild, moderate and severe. The correlation analysis we carried out in our study determined no correlation between sPAP and the parameters evaluated in cases who had bleeding (p>0.05). The reason for this may be the change in clinical parameters, which is an indirect reflection of the pressure decrease in the portal system due to bleeding.

The MELD score was higher in the PHT group compared to those without PHT (p=0.007). This situation can be interpreted as an indicator of negative effects of cardiac functions on the liver and worsening the disease prognosis.

The limiting factor of the study is that the pulmonary artery is not measured by right heart catheterization, which is the gold standard method.

## Conclusions

The prevalence of PHT has increased in patients with PH. Although no significant relationship was found with EV bleeding, it should be noted that the increase in the MELD score, which is an indicator of mortality and morbidity, may be associated with PHT and thus, the patients should be closely monitored for treatment at the appropriate time. Prospective studies using the right heart catheterization are needed to clarify the relationship between PHT and esophageal varice bleeding.
